# Developmental Toxicity and Apoptosis in Zebrafish: The Impact of Lithium Hexafluorophosphate (LiPF_6_) from Lithium-Ion Battery Electrolytes

**DOI:** 10.3390/ijms25179307

**Published:** 2024-08-28

**Authors:** Boyu Yang, Luning Sun, Zheng Peng, Qing Zhang, Mei Lin, Zhilin Peng, Lan Zheng

**Affiliations:** 1 Key Laboratory of Physical Fitness and Exercise Rehabilitation of Hunan Province, Hunan Normal University, Changsha 410012, China; 2 College of Fisheries, Hunan Agricultural University, Changsha 410128, China; 3 The Center for Heart Development, College of Life Sciences, Hunan Normal University, Changsha 410081, China

**Keywords:** lithium-ion batteries, LiPF_6_, developmental toxicity, zebrafish, apoptosis, transcriptomic analysis

## Abstract

With the growing dependence on lithium-ion batteries, there is an urgent need to understand the potential developmental toxicity of LiPF_6_, a key component of these batteries. Although lithium’s toxicity is well-established, the biological toxicity of LiPF_6_ has been minimally explored. This study leverages the zebrafish model to investigate the developmental impact of LiPF_6_ exposure. We observed morphological abnormalities, reduced spontaneous movement, and decreased hatching and swim bladder inflation rates in zebrafish embryos, effects that intensified with higher LiPF_6_ concentrations. Whole-mount in situ hybridization demonstrated that the specific expression of the swim bladder outer mesothelium marker anxa5b was suppressed in the swim bladder region under LiPF_6_ exposure. Transcriptomic analysis disclosed an upregulation of apoptosis-related gene sets. Acridine orange staining further supported significant induction of apoptosis. These findings underscore the environmental and health risks of LiPF_6_ exposure and highlight the necessity for improved waste management strategies for lithium-ion batteries.

## 1. Introduction

In recent decades, there has been a sustained increase in the need for batteries as portable energy sources, a trend largely driven by advances in electronic technology, consumer-oriented design innovations, and the ubiquity of mobile devices [1]. Characterized by compact size, elevated cell voltage, minimal self-discharge, superior energy density, and a broad operational temperature spectrum, lithium-ion batteries have become indispensable across a spectrum of applications, from portable electronics to electric vehicles, as well as in military and space exploration, thereby substantially decreasing reliance on fossil fuels [2,3]. The Chinese lithium-ion battery industry experienced a notable expansion in 2023, with an annual production exceeding 940 GWh, reflecting a 25% growth compared to the previous year and an industry output value surpassing USD 190 billion [4]. Integral to lithium-ion batteries, the electrolyte facilitates lithium-ion transfer across the anode and cathode, predominantly consisting of solvents, additives, and lithium salts [5]. Owing to its exceptional electrical conductivity and high solubility in organic solvents, lithium hexafluorophosphate (LiPF_6_) emerges as the predominant choice for lithium salts in the electrolyte of contemporary liquid-state lithium-ion batteries [6,7,8]. The operational concentration of LiPF_6_ typically ranges from 0.8 to 2 M [7,8].

Landfills remain a prevalent method for managing municipal solid waste (MSW) globally [9,10,11]. A significant proportion of this waste, estimated at 4%, comprises electronic waste (e-waste), which frequently includes batteries. Considering the low recycling rates for lithium-ion batteries, it is reasonable to deduce that a considerable amount of e-waste containing these batteries is likely disposed of in landfills instead of being recycled [12]. Spent lithium-ion batteries contain heavy metal compounds and LiPF_6_, which are recalcitrant to microbial degradation [13]. Improper disposal of these batteries can lead to soil and groundwater contamination, posing significant environmental challenges [13,14].

Lithium has been associated with a broad spectrum of toxic effects in humans, encompassing the nervous system [15], renal function [15,16,17], cardiovascular system [17,18], gastrointestinal tract [17], and endocrine system [17,19,20], as well as reproductive and developmental health [21]. While the toxicity of lithium has been extensively studied, the specific effects of LiPF_6_ exposure, particularly its developmental toxicity, remain less explored. A recent genome-wide screen in yeast has demonstrated that LiPF_6_ is more toxic than lithium chloride (LiCl) or sodium hexafluorophosphate (NaPF_6_), underscoring the need for further investigation into its biological impacts [22]. Given the ubiquitous use of lithium-ion batteries and the consequent risk of LiPF_6_ exposure, an investigation into its developmental impact is both timely and imperative. On the basis of the aforementioned studies, our study transitions to the zebrafish model, a vertebrate system. Zebrafish, with their genetic similarity to humans and transparent embryonic development, serve as an efficient model for toxicological studies, facilitating an examination of the developmental toxicity effects of LiPF_6_ [23,24,25,26].

## 2. Results

### 2.1. Developmental Abnormalities Induced by LiPF_6_ in Zebrafish

**Figure 1:**
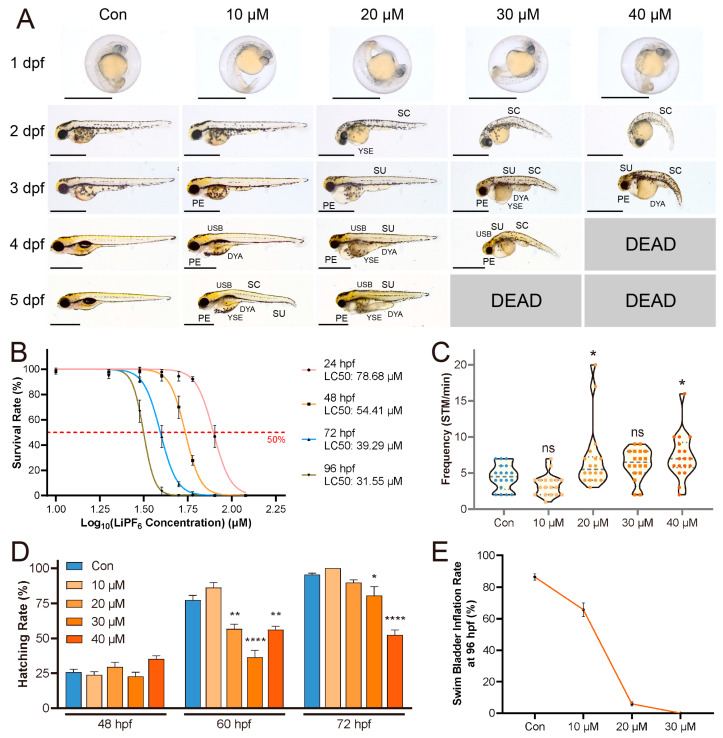
Developmental toxicity of LiPF_6_ in zebrafish embryos. (**A**) Light microscopy images at 1–5 dpf showing the range of malformations in embryos exposed to different concentrations of LiPF_6_, including spinal curvature (SC), pericardial edema (PE), yolk sac edema (YSE), skin ulceration (SU), delayed yolk absorption (DYA) and uninflated swim bladder (USB). The scale bar in each image represents 1 mm; (**B**) LC50 curves at 24, 48, 72, and 96 hpf, showing the lethal concentration of LiPF_6_ that results in 50% mortality in the exposed embryos; (**C**) STM frequency per minute for embryos; (**D**) hatching rates at 48, 60, and 72 hpf; (**E**) swim bladder-inflation rates at 96 hpf. Statistical significance between the exposure groups and Con is indicated by: * (*p* < 0.05), ** (*p* < 0.01), and **** (*p* < 0.0001).

To ascertain the developmental impact of LiPF_6_, this study documents the phenotypic changes in zebrafish, highlighting the early stages of exposure and the associated anomalies. Light microscopy images revealed that from 2 days post fertilization (dpf) onwards, zebrafish embryos exposed to LiPF_6_ exhibited a range of developmental abnormalities that became more pronounced with time and increasing concentrations. The spectrum of malformations included spinal deformities, edema in the pericardial and yolk-sac regions, skin ulceration, and impaired swim bladder inflation (Figure 1A). The LC50 values decreased from 78.68 µM at 24 h post fertilization (hpf) to 31.55 µM at 96 hpf, reflecting the cumulative toxicity of continuous LiPF_6_ exposure rather than an increased sensitivity of the embryos at later stages of development (Figure 1B). Spontaneous tail movement (STM) frequency was found to be higher in the 20 and 40 µM LiPF_6_ groups, compared to the control (Con) (Figure 1C). This increase was accompanied by occasional intense spasms in some embryos, suggesting that these concentrations of LiPF_6_ may induce hyperactivity in zebrafish embryos, although the underlying mechanism remains unclear. Hatching rates were initially similar across all groups at 48 hpf (Figure 1D). However, by 60 hpf, a significant reduction in hatching rates was observed in the 20-to-40 µM groups (Figure 1D). This reduction persisted at 72 hpf, where the hatching rates in the 30 and 40 µM groups remained significantly lower than the control, indicating a sustained effect of LiPF_6_ exposure on hatching success (Figure 1D). At 96 hpf, the swim bladder-inflation rate revealed a marked decrease with escalating LiPF_6_ concentrations, indicating a direct correlation between exposure levels and the impairment of this developmental parameter (Figure 1E). The determination of swim bladder-inflation status was based on microscopic observation (Figure S1).

### 2.2. LiPF_6_-Induced Downregulation of anxa5b in Zebrafish Swim Bladder Development

**Figure 2:**
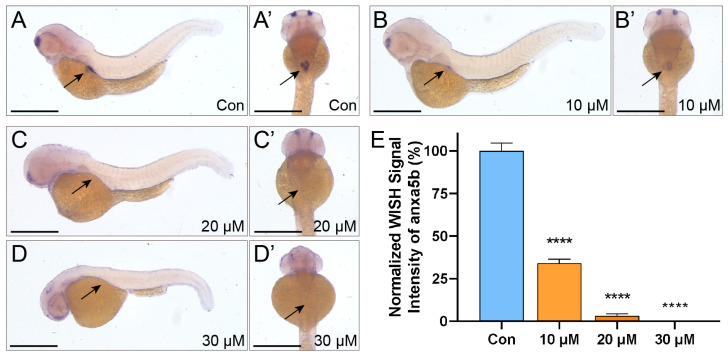
WISH Analysis of anxa5b in 60 hpf zebrafish exposed to LiPF_6_. (**A**–**D**) Representative WISH images of anxa5b expression of 60 hpf zebrafish. Panels show embryos exposed to increasing concentrations of LiPF_6_: control (**A**), 10 µM (**B**), 20 µM (**C**), and 30 µM (**D**). Corresponding dorsal views, denoted as (**A′**–**D′**). The scale bar in each image represents 1 mm; (**E**) quantitative analysis of WISH signal intensity in the swim bladder mesothelium (n = 6 per group). Statistical significance between the exposure groups and Con is indicated by: **** (*p* < 0.0001).

Following the observation of reduced swim bladder-inflation rates at 96 hpf in zebrafish embryos exposed to LiPF_6_, we further investigated the molecular underpinnings of this phenotype by examining the expression of anxa5b. This outer mesothelium marker of the swim bladder begins to express at 60 hpf [27,28]. Whole-Mount In Situ Hybridization (WISH) for anxa5b was conducted on embryos at 60 hpf to assess the impact of LiPF_6_ exposure on this critical developmental process. In control embryos (Con), the swim bladder at the growth phase displayed a robust anxa5b signal, indicative of active tissue differentiation (Figure 2A,A′). However, this signal intensity was significantly diminished in a concentration-dependent manner upon exposure to LiPF_6_ at 10–30 µM (Figure 2B–D,B′–D′). Notably, at 20 µM LiPF_6_ exposure, the anxa5b signal in the swim bladder region was significantly diminished, becoming barely discernible (Figure 2C,C′). At the highest concentration tested, 30 µM, the signal in the swim bladder was nearly absent, suggesting a near-complete inhibition of anxa5b expression at these concentrations (Figure 2D,D′). Quantitative analysis revealed the most pronounced reduction observed at the 30 µM (Figure 2E). The concentration-dependent downregulation of anxa5b expression in the swim bladder at the growth phase correlates with the observed reduction in swim bladder-inflation rates, suggesting that LiPF_6_ exposure may impair the differentiation of the swim-bladder outer mesothelium, a process essential for proper swim bladder development.

### 2.3. Molecular Signatures of LiPF_6_ Toxicity Unveiled through Transcriptomic Profiling

**Figure 3:**
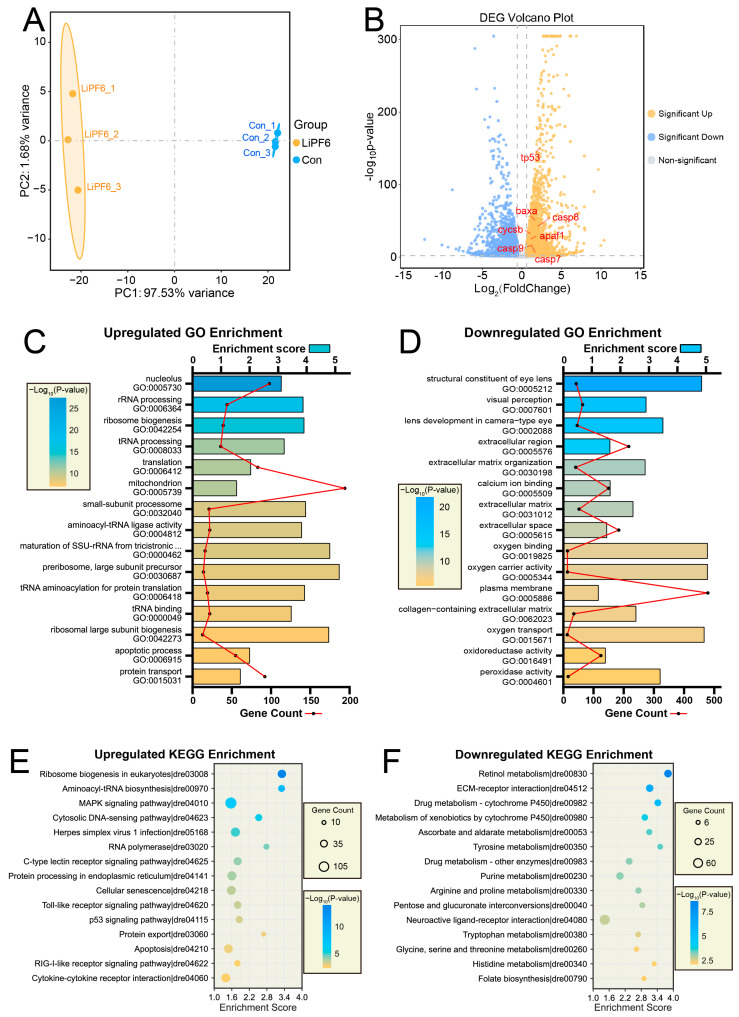
Transcriptomic analysis of zebrafish exposed to LiPF_6_ at 48 hpf. (**A**) Principal component analysis (PCA) plot of the transcriptome sequencing data from control and LiPF_6_-exposed (40 µM) zebrafish at 48 hpf; (**B**) volcano plot depicting the DEGs identified from the transcriptome sequencing analysis, with the DEGs identified with a *p*-value < 0.01 and an absolute fold change > 1.5 (log_2_ scale). Pro-apoptotic genes are highlighted, all of which are upregulated DEGs; (**C**,**D**) combined dual-axis bar-line charts for GO enrichment analysis of both upregulated (**C**) and downregulated (**D**) DEGs. These charts display the top-15 enriched terms ranked by ascending *p*-value. The bars represent enrichment scores on the upper axis, while the line traces gene counts on the lower axis. Bar coloration indicates the *p*-value of the enrichment; (**E**,**F**) KEGG enrichment-analysis bubble charts for upregulated (**E**) and downregulated (**F**) DEGs, displaying the top-15 pathways ranked by ascending *p*-value.

In light of the earliest morphological phenotypes observed at 2 dpf, we planned an RNA-Seq analysis for the same stage to offer a thorough and systematic assessment of the molecular alterations from 40 µM LiPF_6_ exposure in zebrafish. The principal component analysis (PCA) exhibited a distinct separation between the control and treated groups (Figure 3A), indicative of a substantial transcriptional reprogramming in response to LiPF_6_. Genes with a *p*-value less than 0.01 and an absolute fold change greater than 1.5 were considered differentially expressed genes (DEGs), as visualized in the volcano plot (Figure 3B). Among the DEGs, the upregulation of pro-apoptotic genes like tp53 and casp7 (Figure 3B) hinted at an apoptotic response to LiPF_6_. Kyoto Encyclopedia of Genes and Genomes (KEGG) and Gene Ontology (GO) enrichment analyses were conducted to further dissect the functional categories and biological pathways affected by the exposure. Notably, the GO enrichment analysis (Figure 3C) for upregulated DEGs prominently features the apoptosis process, underscoring the activation of biological pathways integral to programmed cell death. In concert, the KEGG pathway analysis (Figure 3E) identifies the apoptosis pathway as significantly enriched among these DEGs, indicating a convergence of molecular signals that orchestrate this critical cellular process. The coordinated upregulation observed across both functional and pathway-based enrichments suggests a unified apoptotic response to LiPF_6_ exposure, highlighting the pertinence of these pathways in mediating the observed phenotypic changes. The downregulated DEGs also showed enrichment in biological processes and pathways, as indicated by the GO (Figure 3D) and KEGG (Figure 3F) enrichment charts, which presented the top-15 enriched terms ranked by *p*-value, offering a broader view of the affected cellular functions. These transcriptomic findings provide a comprehensive view of the molecular response to LiPF_6_ exposure, laying the groundwork for further exploration of the mechanisms involved in the developmental effects observed.

**Figure 4:**
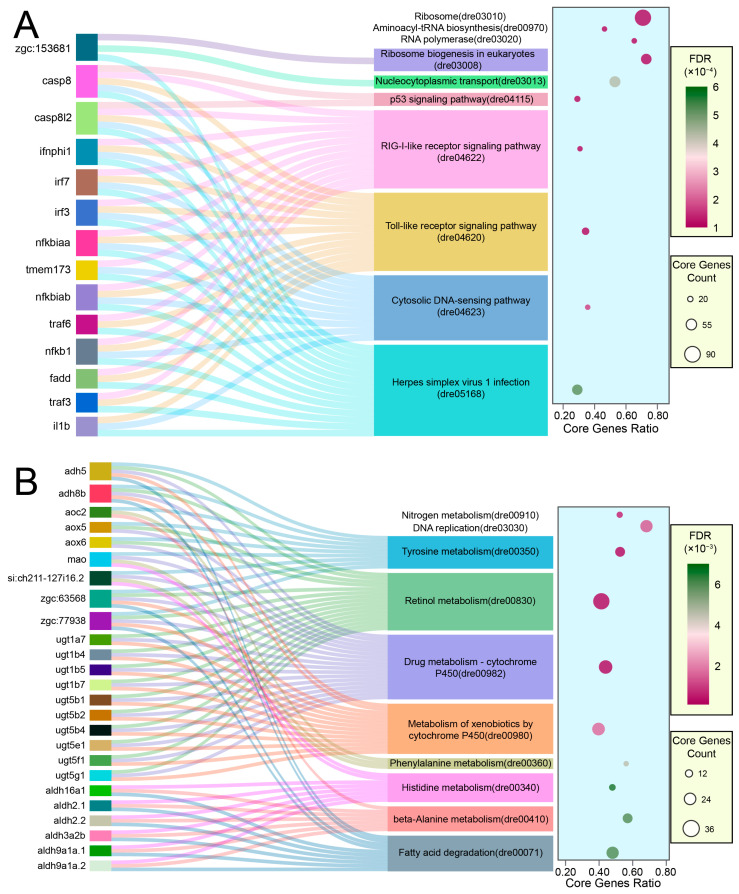
GSEA-KEGG enrichment landscape induced by LiPF_6_ exposure. This figure employs a Sankey bubble plot to trace the interplay of core genes across the top-ten upregulated (**A**) and downregulated (**B**) GSEA-KEGG terms, ranked by their |NES| from the GSEA-KEGG analysis. The chart highlights genes that are common to at least three pathways, signifying their key roles in the cellular response to LiPF_6_. The Sankey diagram delineates these connections, while the juxtaposed bubble chart provides a quantitative assessment of the enrichment, reflecting the FDR, core gene count, and core gene ratio for each pathway.

The Sankey bubble plot visually maps the multi-pathway engagement of core genes, as revealed by Gene Set Enrichment Analysis (GSEA)–KEGG analysis, to offer a detailed perspective on the gene expression changes induced by LiPF_6_ exposure (Figure 4). The upregulation observed in GSEA-KEGG enriched terms, signified by high normalized-enrichment scores (NES), points to the activation of pathways such as the “p53 signaling pathway” (dre04115) (Figure 4A). Notably, the genes casp8 and casp8l2, integral components of the apoptotic machinery, are the most interconnected nodes across the enriched pathways, including immune-related pathways such as the “RIG-I-like receptor signaling pathway” (dre04622), “Toll-like receptor signaling pathway” (dre04620), “Cytosolic DNA-sensing pathway” (dre04623), “Herpes simplex virus 1 infection” (dre05168) and the “p53 signaling pathway” (dre04115) (Figure 4A). These interconnected pathways suggest a coordinated response to stress that encompasses both immune activation and the potential initiation of apoptotic mechanisms. The downregulated gene set, characterized by a high absolute NES, predominantly implicates metabolic pathways, including “Nitrogen metabolism” (dre00910) and “Drug metabolism—cytochrome P450” (dre00982), indicating a reconfiguration of cellular metabolism under stress (Figure 4B). The presence of genes shared across multiple pathways in both the upregulated and downregulated sets indicates a complex interplay of biological processes, with apoptosis emerging as a central theme of the cellular response to LiPF_6_.

### 2.4. Zebrafish Exhibit a Pronounced Apoptotic Response upon LiPF_6_ Exposure

**Figure 5:**
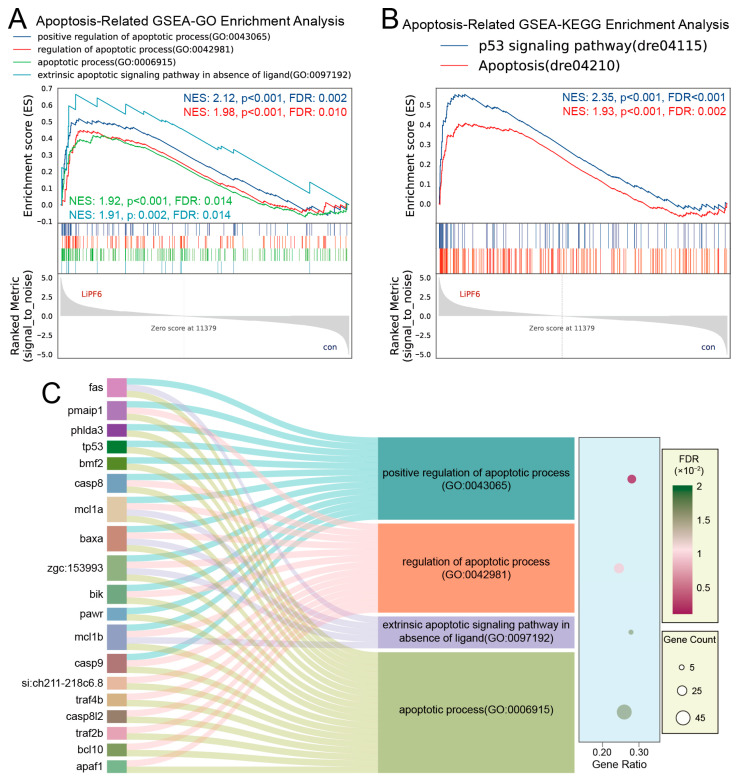
GSEA of apoptosis-related gene sets in zebrafish exposed to LiPF_6_. (**A**) Enrichment plots for four GSEA-GO terms related to apoptosis; (**B**) Enrichment plots for two KEGG-GSEA terms associated with apoptosis; (**C**) A Sankey bubble plot depicting the overlap of genes across the enriched GO terms, highlighting genes retained in two or more sets.

The GSEA-GO analyses related to the regulation of apoptosis were significantly enriched, indicating an orchestrated activation of apoptosis pathways in response to LiPF_6_ exposure (Figure 5A). Similarly, the GSEA-KEGG terms identified, including the p53 signaling pathway and apoptosis pathway, were also significantly enriched, underscoring the centrality of these pathways in the apoptotic response (Figure 5B). Among the six apoptosis-related GSEA terms identified, each displayed a normalized enrichment score (NES) hovering around 2, a notably high value that signals a substantial biological effect. The concurrence of *p*-values ≤ 0.002 and FDR ≤ 0.014 substantiates the significant upregulation of these pathways upon LiPF_6_ exposure (Figure 5A,B). This robust enrichment indicates that the apoptotic response is not only enriched, but is likely a major contributor to the toxicological profile of LiPF_6_ in zebrafish. To further elucidate the molecular underpinnings of these enrichments, a Sankey bubble plot was employed to depict the intersection of core genes involved in the enriched GO terms (Figure 5C). This visualization revealed a subset of genes that were consistently implicated across multiple gene sets, pointing to a core apoptotic gene signature in response to LiPF_6_ exposure.

**Figure 6:**
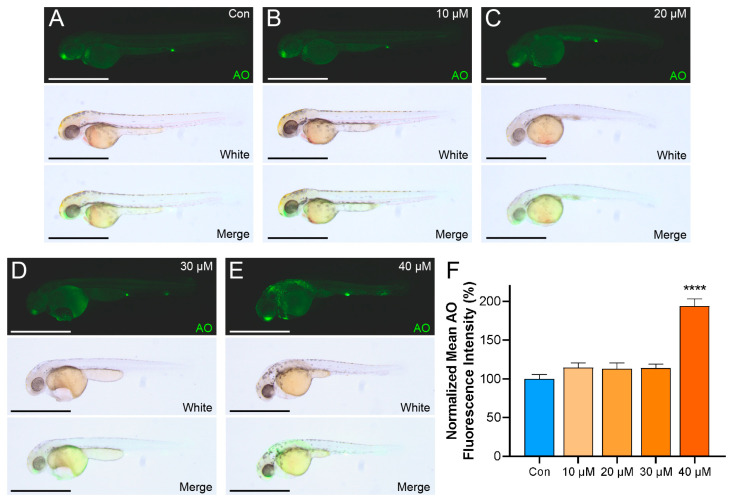
AO staining indicates apoptotic induction in 2 dpf zebrafish by LiPF_6_ exposure. (**A**–**E**) AO-staining images of zebrafish larvae exposed to increasing concentrations of LiPF_6_: (**A**) control, (**B**) 10 µM, (**C**) 20 µM, (**D**) 30 µM, and (**E**) 40 µM. Each panel is annotated with “AO” for fluorescence, “White” for brightfield, and “Merge” for the overlay of fluorescence and brightfield images. The scale bar in each image represents 1 mm; (**F**) quantitative analysis of AO-fluorescence intensity normalized to the control group (n ≥ 5 per group). Statistical significance between the exposure groups and Con is indicated by: **** (*p* < 0.0001).

Building on the transcriptomic findings, Acridine Orange (AO) staining served as an initial approach to examine the potential apoptotic effects in zebrafish treated with LiPF_6_. In groups treated with 10 µM (Figure 6B), 20 µM (Figure 6C), and 30 µM (Figure 6D), a subtle enhancement of AO fluorescence was recorded in contrast to the control (Figure 6A). This enhancement was especially pronounced in regions encircling the edematous pericardium and yolk sac, where granular fluorescence was observed. This fluorescence increment suggests an inclination towards apoptosis. However, this elevation did not reach statistical significance (Figure 6F). In the 40 µM group (Figure 6E), a more pronounced rise in fluorescence was observed, potentially indicative of an increased apoptotic response. Quantitative fluorescence-intensity measurements support these visual observations, with a significant increase detected at the 40 µM concentration (Figure 6F). In the context of 40 µM LiPF_6_ exposure, zebrafish not only showed characteristic granular fluorescence around the edematous pericardium and yolk sac, but also revealed similar fluorescence in the tail and anterior trunk regions (Figure 6E), which may indicate an expanded apoptotic effect. The findings from AO staining hint at an apoptotic trend in zebrafish subjected to LiPF_6_ exposure, aligning with the transcriptomic evidence pointing towards the activation of apoptosis-related pathways.

**Figure 7:**
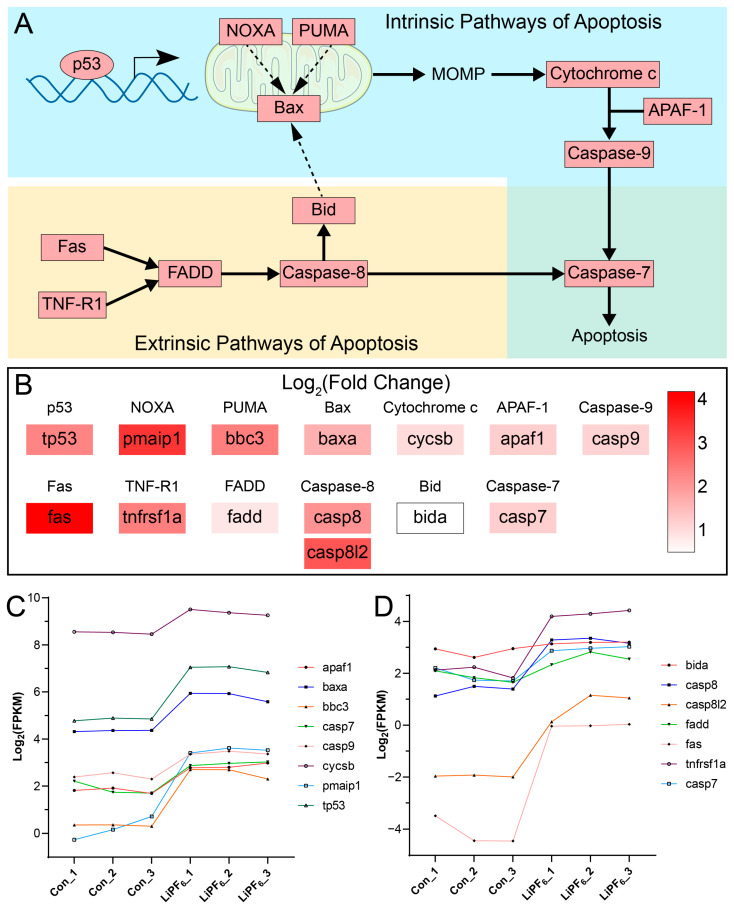
Mechanism of apoptosis induced by LiPF_6_ exposure in zebrafish. (**A**) Schematic of key elements in the apoptotic pathways, highlighting the intrinsic and extrinsic routes converging at caspase-7. All gene-encoded elements are depicted in red, signifying upregulation; (**B**) Log_2_(Fold Change) heatmap of the genes encoding the elements in (Figure 7A), illustrating the upregulation spectrum; (**C**,**D**) line graphs illustrating the Log_2_(FPKM) values of genes in the intrinsic (**C**) and extrinsic (**D**) apoptotic pathways upon LiPF_6_ exposure.

Given the convergence of evidence from both the GSEA analysis and the AO staining, which point towards the activation of apoptosis, a more detailed exploration of the molecular mechanisms underlying apoptosis in LiPF_6_-exposed zebrafish was warranted. We aimed to dissect the signaling pathways that culminate in the activation of caspase-7, a central mediator of apoptosis. In the intrinsic apoptotic pathway, p53 initiates the transcriptional upregulation of NOXA, PUMA, and Bax (Figure 7A) [29]. The upregulation of NOXA and PUMA is posited to indirectly promote Bax activity, a key effector that facilitates mitochondrial outer-membrane permeabilization (MOMP) (Figure 7A) [30,31,32]. The release of cytochrome c, subsequent to MOMP, binds to APAF-1, culminating in the formation of the apoptosome and the activation of caspase-9 (Figure 7A) [33,34,35]. This initiator caspase subsequently engages caspase-7, an executioner caspase that propels the cell towards apoptosis (Figure 7A) [36]. In the extrinsic apoptotic pathway, the activation of death receptors like Fas and TNF-R1 recruits FADD, leading to caspase-8 activation (Figure 7A) [37,38,39]. Caspase-8 then directly activates caspase-7, a convergence point for both extrinsic and intrinsic pathways, thereby streamlining the apoptotic process (Figure 7A) [36,40]. Moreover, caspase-8 cleaves Bid, which in turn activates Bax and induces MOMP, indirectly reinforcing the intrinsic pathway (Figure 7A) [41,42,43]. The heatmap provides a quantitative representation of the upregulation of genes encoding these proteins, as evidenced by the log_2_(Fold Change) values of fragments per kilobase of transcript per million fragments mapped (FPKM) and the corresponding color intensity (Figure 7B). The line graphs further elucidate the trend of gene upregulation in the intrinsic (Figure 7C) and extrinsic (Figure 7D) pathways, respectively, in response to LiPF_6_ exposure.

## 3. Discussion

The current study provides a comprehensive examination of the developmental toxicity of LiPF_6_, a ubiquitous component of lithium-ion batteries, using the zebrafish model. Our findings of increased developmental abnormalities with escalating LiPF_6_ concentrations underscore the environmental and health risks associated with improper disposal of lithium-ion batteries.

The hexafluorophosphate (PF_6_^−^) anion is known for its susceptibility to hydrolysis in electrolyte environments, a phenomenon extensively documented in the experimental and theoretical literature [44,45,46]. Beyond the scope of battery applications, PF_6_^-^ is a commonly used anion in chemistry, with its compounds demonstrating relative stability in aqueous solutions [47]. Studies have shown that LiPF_6_ exhibits minimal hydrolysis in water, with no significant degradation observed even after three weeks of dissolution [48,49]. Given the absence of organic solvents in environments where LiPF_6_ may leak, the chemical stability of LiPF_6_ is likely to increase. This highlights the necessity to investigate the toxicological profile of LiPF_6_ under such conditions. To minimize potential interference from trace hydrolysis products such as hydrogen fluoride (HF) and dihydrogen phosphite (HPO_2_F_2_) in our toxicological studies [48], we have implemented strict protocols. Our solutions of LiPF_6_ in water are prepared to be used within 24 h, stored at 4 °C, and refreshed daily for zebrafish exposure, to ensure the integrity of our experimental conditions.

The swim bladder’s inability to inflate, or the reduction in inflation rates, have been widely documented as a response to various stress factors during zebrafish development, including genetic mutations, exposure to toxicants, temperature fluctuations, osmotic stress, pH imbalances, and signaling pathway disruptions [50,51,52,53]. This response appears not to be specific to any single stressor, but rather represents a complex physiological reaction to environmental and intrinsic challenges. The swim bladder is a complex organ composed of three distinct tissue layers: epithelium, mesenchyme, and mesothelium [27,28,54]. The anxa5b gene, serving as a marker for the outer mesothelium, is not exclusively expressed in the swim bladder. It is also found in other regions, such as the nose and the proctodeum. However, the use of WISH allows for the specific observation and quantification of anxa5b expression changes in the swim bladder region, without interference from its expression in other tissues. This technique provides a unique advantage in assessing the localized impact of LiPF_6_ on anxa5b expression, offering insights into the molecular mechanisms that may underlie the observed swim-bladder developmental anomalies.

The Sankey bubble plots employed in our transcriptomic analysis provide a sophisticated visualization of the interplay between DEGs and their associated pathways. By retaining DEGs that recur across multiple enriched pathways, the Sankey component of the plot highlights the connectivity of these genes to their respective pathways, underscoring the multifunctional nature of DEGs. Concurrently, the bubble chart element conveys a rich dataset of enrichment metrics, such as significance, gene count, and enrichment scores, offering a quantitative perspective on the biological impact of these genes. This dual representation not only deepens our understanding of the hierarchical organization of pathways, but also aids in pinpointing key regulatory genes and potential biomarkers that are critical for deciphering the molecular underpinnings of the observed phenotypes.

Our transcriptomic analysis has uncovered a nuanced molecular response to LiPF_6_ exposure, revealing a pro-apoptotic mechanism. The observed upregulation of genes pivotal to the apoptotic process, such as tp53 and casp7, points towards an intensified apoptotic response. This response could be central to the developmental irregularities witnessed in zebrafish embryos exposed to LiPF_6_. Moreover, our data indicate a transcriptional upregulation of the NF-κB signaling pathway, typically associated with cell survival and inflammation. While this upregulation does not directly suggest pathway activation, it is indicative of a potential counter-response to the apoptotic stimuli. The transcriptional changes observed in the NF-κB pathway may reflect an adaptive cellular mechanism aimed at mitigating the apoptotic impact of LiPF_6_, adding a layer of complexity to the embryo’s stress response. Given the diffuse and widespread nature of apoptosis during embryogenesis, the quantification of AO staining in our study was performed by measuring the average fluorescence intensity across the entire zebrafish embryo. This approach ensures a comprehensive assessment of apoptotic events. AO staining is recognized for its high specificity for apoptotic cells within whole live embryos [55,56,57]. Although the exact mechanisms underlying this selectivity are not fully elucidated, it is believed that AO’s spectral-emission properties change as cellular proteins undergo modifications during the condensation of apoptotic corpses [56].

Although speculative, the activation of the apoptotic pathway by LiPF_6_ may involve direct interactions with cellular components or indirect effects through the disruption of cellular signaling networks. For instance, LiPF_6_ could potentially alter mitochondrial function, leading to the generation of reactive oxygen species (ROS) and the subsequent activation of p53. Further research is necessary to determine the precise molecular mechanisms by which LiPF_6_ induces apoptosis. It will be important to investigate whether LiPF_6_ exposure leads to ROS production, mitochondrial dysfunction, or other cellular changes that could trigger the activation of p53 and the downstream apoptotic cascade.

The findings from this study underscore the environmental risks associated with the disposal of lithium-ion batteries, particularly in aquatic ecosystems. Furthermore, the properties of LiPF_6_ may have broad implications for human health, given the increasing exposure to LiPF_6_ through various consumer products. Our study presents a contribution to the understanding of the developmental toxicity of LiPF_6_, highlighting the need for improved waste-management strategies for lithium-ion batteries and further research into the toxicity of LiPF_6_. The zebrafish model provides a robust platform for toxicological studies aimed at mitigating the environmental and health risks posed by emerging technologies.

## 4. Materials and Methods

### 4.1. Cultivation and Propagation of Zebrafish

For the investigation, mature specimens of the AB strain (a well-established and commonly used zebrafish strain) of zebrafish (*Danio rerio*) were selected from the in-house aquatic facility. The aquatic environment was prepared using reverse-osmosis filtered municipal water, supplemented with NaCl and NaHCO_3_ to achieve a specific conductivity range of 500–700 μS/cm and a pH balance between 6.5 and 7.5. The ambient temperature was regulated at 27 ± 1 °C, and a light regime of 14 h illumination followed by 10 h darkness was implemented to mimic natural circadian rhythms. The breeding pairs were housed in separate tanks, with a 1:1 female-to-male ratio, and the resulting fertilized eggs were nurtured in E3 medium (5.00 mmol/L NaCl, 0.17 mmol/L KCl, 0.33 mmol/L CaCl_2_, and 0.33 mmol/L MgSO_4_).

### 4.2. Embryo Exposure Protocol

Lithium hexafluorophosphate (LiPF_6_, 98% purity) was procured from Maclin, China. Stock solutions were formulated using E3 medium and stored under refrigeration at 4 °C, with reconstitutions performed immediately upon surpassing a 24 h threshold from initial preparation, to ensure solution potency and stability. Based on preliminary experiments that identified an approximate lethal-concentration range for LiPF_6_ in zebrafish embryos, a series of concentrations were selected for detailed dose–response analysis: 0 (control), 10, 20, 30, 40, 50, 60, 80, and 120 µM in E3 medium. The LC50 values were calculated at 24, 48, 72, and 96 hpf, using GraphPad Prism software (version 8.0, GraphPad Software, San Diego, CA, USA), employing a dose–response inhibition model. Following the determination of the LC50, further experiments were narrowed down to concentrations of 10, 20, 30, and 40 µM LiPF_6_, with an additional con (0 µM LiPF_6_) group, to investigate the developmental toxicity in detail. Zebrafish embryos were screened at 6 hpf to exclude any with developmental irregularities, ensuring that only healthy embryos were used for exposure. A total of 30 embryos were allocated to each concentration group, with each group containing at least 3 replicates, to ensure the robustness of the results. Exposures were conducted under stable conditions at 28 ± 1 °C for up to 5 dpf. The exposure was carried out in 100 mL glass containers, each containing 50 mL of exposure solution, with the medium refreshed every 24 h to ensure stable and consistent conditions.

### 4.3. Assessment of LiPF_6_ Toxicity in Zebrafish Development

At 1, 2, 3, and 4 dpf, LC50 values were determined for LiPF_6_ by plotting survival against the log10-transformed concentrations. The LC50 was calculated using GraphPad Prism software, which fits the data to a dose–response inhibition model. At the 24 hpf, the STM frequency of the embryos was documented using a stereomicroscope, with each experimental group comprising 18 embryos. Developmental anomalies were documented through photographic records between 1 and 5 dpf. Assessments of the survival rate, hatching rate, and swim bladder-inflation rate were conducted with triplicate replicates, with each replicate consisting of 30 embryos.

### 4.4. Whole-Mount In Situ Hybridization

To determine the expression of the anxa5b gene, particularly within the developing swim bladder anlage, WISH was employed on zebrafish embryos. A DNA template specific to the anxa5b gene was PCR-amplified using the following primers: Forward Primer 5′-TGCTGTTTTGCTACAGGCCA-3′ and Reverse Primer 5′-GCGTAATACGACTCACTATAGGGCTCAGTCGGGTATAGAGGTGGA-3′. The antisense RNA probe was synthesized from the PCR product using T7 RNA polymerase, incorporating digoxigenin (DIG) as a label. Hybridization and detection were performed according to an established protocol [58]. The total intensity of the specific hybridization signal in the swim bladder was quantified using ImageJ (version 1.54f).

### 4.5. RNA-Seq and Analysis

Zebrafish embryos developing to 48 hpf were prepared for exposure to 40 µM LiPF_6_ and a control group, with three biological replicates per group, each containing 50 ± 2 embryos, was prepared. Surviving embryos were collected for RNA extraction. The quality of the extracted RNA was verified using a NanoDrop2000 spectrophotometer (ThermoFisher, Waltham, MA, USA), ensuring an optical density (OD) ratio of 260/280 ≥ 1.8, and 260/230 ≥ 2.0. RNA integrity was further confirmed with an Agilent bioanalyzer, with samples required to have an RNA integrity number (RIN) of ≥9. cDNA libraries were synthesized from the purified RNA and sequenced on the Illumina platform. Raw sequencing reads underwent quality control using RseQC [59] (version 4.0.0) and FastQC (version 0.11.9), resulting in a set of clean reads. These clean reads were aligned to the zebrafish genome reference sequence (GRCz11) using Hisat2 (version 2.1.0). The read counts were normalized and analyzed for differential gene expression using the DESeq2 R package (version 1.22.2), identifying genes as DEGs with a *p*-value < 0.01 and an absolute fold change > 1.5. GO and KEGG enrichment analyses were conducted on the DEGs. The hypergeometric distribution test was applied to calculate the significance of enrichment for each category. GSEA was conducted using the “signal-to-noise” method to analyze the enrichment of gene sets from the GO and KEGG databases.

### 4.6. Assessment of Apoptosis in Zebrafish Embryos via AO Staining

Apoptosis in zebrafish embryos was assessed using AO staining, based on a method detailed in a prior publication [60]. Post staining, embryos were anesthetized with MS-222, prior to imaging. Green fluorescence, indicative of AO staining, was visualized under a fluorescence stereomicroscope (Leica, Wetzlar, Germany) equipped with a GFP filter set. The mean green-fluorescence intensity of the zebrafish samples was quantified using ImageJ software. This quantification involved measuring the mean fluorescence intensity of the samples and the background separately, followed by subtracting the background mean from the sample mean to obtain the corrected mean AO green-fluorescence intensity.

### 4.7. Statistical Analysis

Data for a single group with multiple replicates are depicted as Mean ± SEM in the figures. For the bar and violin plots, one-way ANOVA followed by Dunnett’s post hoc test was utilized to evaluate the significance of differences exclusively between the various LiPF_6_ exposure concentration groups and the Con group. The significance levels are represented as ns (*p* ≥ 0.05), * (*p* < 0.05), ** (*p* < 0.01) and **** (*p* < 0.0001).

## Data Availability

Dataset available on request from the authors.

## Supplementary Materials

The following supporting information can be downloaded at: https://www.mdpi.com/article/10.3390/ijms25179307/s1.
