# Supplementary figures and images for: Developmental Toxicity and Apoptosis in Zebrafish: The Impact of Lithium Hexafluorophosphate (LiPF6) from Lithium-Ion Battery Electrolytes
Source: Int J Mol Sci. 2024 Aug 28;25(17):9307. doi: 10.3390/ijms25179307 (PMC11395654; doi:10.3390/ijms25179307)

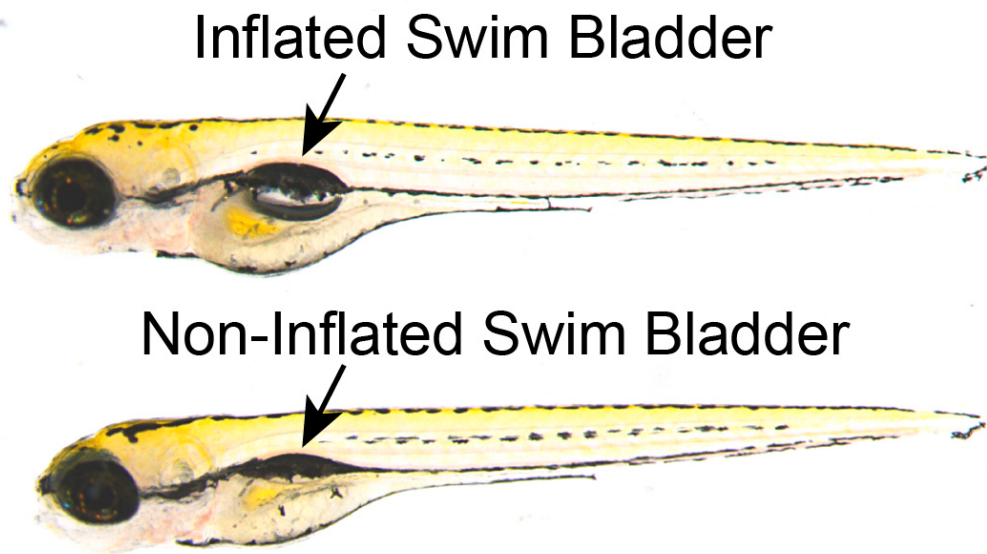

**Figure S1.** Determination of Swim Bladder in Zebrafish

Supplement: Supplementary file 1 [file ijms-25-09307-s001.zip › ijms-3150488-supplementary.pdf]
